# Built and natural environment planning principles for promoting health: an umbrella review

**DOI:** 10.1186/s12889-018-5870-2

**Published:** 2018-07-28

**Authors:** E. L. Bird, J. O. Ige, P. Pilkington, A. Pinto, C. Petrokofsky, J. Burgess-Allen

**Affiliations:** 1 Faculty of Health and Applied Sciences, University of the West of England, Frenchay Campus, Bristol, BS16 1QY UK; 2 Health Equity & Mental Health Division, Health Improvement Directorate, Public Health England, 2nd Floor Skipton House, 80 London Road, London, SE1 6LH UK; 3 Public Health, Derby City Council, 1st Floor, The Council House, Corporation Street, Derby, DE1 2FS UK

**Keywords:** Umbrella review, Built and natural environment, Health, Planning

## Abstract

**Background:**

The built and natural environment and health are inextricably linked. However, there is considerable debate surrounding the strength and quality of the evidence base underpinning principles of good practice for built and natural environment design in promoting health. This umbrella review aimed to assess relationships between the built and natural environment and health, concentrating on five topic areas: neighbourhood design, housing, food environment, natural and sustainable environment, and transport.

**Methods:**

A structured search was conducted for quantitative systematic reviews and stakeholder reviews published between January 2005 and April 2016. Seven databases and the websites of 15 relevant and respected stakeholder organisations known to publish review-level documentation were searched. Searches were limited to English-language publications and duplicate references were removed. Evidence quality and strength was appraised using validated techniques. Findings were used to develop a diagram for each topic area, illustrating relationships between built and natural environment planning principles and health-related outcomes.

**Results:**

A total of 117 systematic reviews and review-level documents were eligible for inclusion. The quality of evidence was mixed; much of the evidence examined relied on findings from cross-sectional studies, making it difficult to draw clear causal links between built environment exposures and health-related impacts and outcomes. Fourteen actionable planning principles associated with positive health-related outcomes were identified across the five topic areas. For example, neighbourhoods that enhanced walkability, were complete and compact in design, and those which enhanced connectivity through safe and efficient infrastructure were associated with better health-related outcomes relating to physical activity, social engagement, mental health, perceptions of crime, and road traffic collisions. Evidence for the effectiveness of planning principles across different topic areas and on reducing health inequalities was sparse and inconclusive.

**Conclusions:**

Findings provide an up-to-date overview of relationships between the built and natural environment and health and present logical, evidence-based messages to aid communication between public health and planning professionals.

**Electronic supplementary material:**

The online version of this article (10.1186/s12889-018-5870-2) contains supplementary material, which is available to authorized users.

## Background

The built and natural environment encompasses objective and subjective features of the physical environment in which people live, work and play, [1] and there is a considerable body of evidence linking the built and natural environment with health and wellbeing [2, 3]. As such, public health and planning professionals are increasingly encouraged to consider the built and natural environment as an important determinant of health [3].

Establishing a causal relationship between built and natural environment characteristics and health outcomes is not easy. Much of the evidence to date relies on findings from observational epidemiological studies which highlight associations between the built and natural environment and health. However, the broadly cross-sectional nature of the evidence base means it is often difficult to determine cause and effect relationships between built environment exposures and health-related impacts and outcomes, and the potential effectiveness of interventions [2, 4]. It has also been acknowledged that evidence for the effectiveness of built environment interventions on tackling health inequalities is limited and unclear [2].

The translation of research evidence into practice has been hindered by the sheer complexity of relationships between the built environment and health, in that they are both influenced by numerous, and sometimes conflicting factors [5], and the methods used to assess these relationships are rarely longitudinal [6]. These factors make it difficult to develop approaches that can be universally applied [5].

Despite these challenges, the volume of literature published in the last decade on links between the built and natural environment and health cannot be ignored. There is a need to take stock of the existing evidence base to consolidate our understanding and, where appropriate, to make recommendations to support those working in public health and planning professions. It was in response to this need that in January 2016 Public Health England (PHE) commissioned this umbrella review. The emergence of umbrella reviews in recent years has provided an attractive strategy for organising and assessing a wide range of review-level evidence [7, 8]. The approach is increasingly used in public health research and practice, bringing together a wide range of evidence to explore what is known about a topic in an attempt to guide the decisions of policy makers [2, 9, 10]. Through this umbrella review we aimed to assess the relationships between the built and natural environment and health, concentrating on five topic areas: neighbourhood design; housing; food environment; natural and sustainable environment; and, transport. The topic areas were defined by the funders, PHE in response to a previous review [11]. PHE colleagues reviewed the Canadian publication which seemed to have a resonance with the focus of their work in this area connecting planning and public health in addressing key issues.

## Methods

### Search strategy

A structured search of the Cochrane Database of Systematic Reviews, EPPI-Centre, MEDLINE, PsycINFO, SafetyLit, Transport Research Information Service, and Applied Social Sciences Index and Abstracts was conducted in April 2016 to identify quantitative systematic reviews (Table 1). Given the practice-based focus of this umbrella review, and in accordance with previous umbrella reviews, [10] we also manually searched the websites of fifteen relevant and respected stakeholder organisations for review-level evidence on the built and natural environment and health (referred to as ‘stakeholder documentation’ from this point on) (see Additional files 1, 2 and 3). Reference lists of eligible reviews were searched, and contact with experts working in the built and natural environment and health fields was initiated to identify documentation not identified through the database search. Search terms were adapted from recent systematic reviews examining aspects of the built environment and health, [3, 12, 13] and were categorised according to built and natural environment characteristics, health-related impacts and/or outcomes and study type.

**Table 1:** Search strategy for electronic databases

Built environment characteristics	Built environment OR neighbourhood design OR housing OR healthy food OR natural environment OR sustainable environment OR transport* OR smart growth OR urban planning OR urban environment OR physical environment OR spatial planning OR food availability OR food environment OR open space OR outdoor* OR countryside OR nature OR allotment OR air quality OR air pollution OR construction facility OR design OR planning OR land use mix or residential OR walkability OR traffic OR green space OR social mix OR housing mix OR salutogenic environments OR liveable environments OR urban design OR cycle networks OR cycle provision OR pedestrian provision OR car-free developments OR home zones
	AND
Health outcomes	Health outcome OR health OR health gain* OR injury preven* OR accident OR physical health OR mental health OR emotional health OR blood pressure OR physical activity OR diet OR activ* OR exercise OR nutrition OR energy intake OR obes* or overweight OR fruit and vegetable OR cardiovascular OR CVD OR suicide OR violence OR disorder OR road safety OR wellbeing OR well-being OR disability OR sedent* OR moderate-to-vigorous physical activity OR MVPA or weight status OR walking OR cycling or road traffic collision OR RTC or RTA or alcohol
	AND
Study type	Systematic review OR meta-analys*

Note. * = Truncation. Electronic databases searched: Cochrane Database of Systematic Reviews; EPPI CENTRE; MEDLINE; PsycINFO; SafetyLit; Transport Research Information Service (TRIS); and, Applied Social Sciences Index and Abstracts (ASSIA)

### Inclusion and exclusion criteria

If a review presented findings on more than one of the five built environment categories or on more than one health outcome, these were assessed and reported separately. Reviews reporting on adults and children (of all ages) were considered for inclusion, as were all health-related outcomes (physical and mental). Reviews published between January 2005 and April 2016 and conducted in high- and middle-income countries (Europe, North America, Australasia, and Japan) were eligible. Searches were limited to English-language publications and duplicates were removed. Evidence from qualitative systematic reviews was excluded. In line with previous umbrella reviews, [2, 9, 10] systematic reviews were required to meet the Database of Abstracts of Reviews of Effects criteria: (1) inclusion of defined research question and (2) search strategy including at least one named database, in conjunction with either reference checking, hand searching, citation searching or contact with authors in the field.

### Data extraction

Potentially eligible papers were screened for inclusion by one reviewer (JI) according to title and abstract. A 10% sample of search results was independently assessed by a second reviewer (EB). Full text articles and stakeholder documentation were then obtained and assessed by two reviewers (EB, JI) against the inclusion criteria. Descriptive data were extracted using a data extraction tool (author, year of publication, population of interest, health outcome(s) and key findings).

### Quality appraisal

#### Quality of review-level evidence

The Methodological Quality Checklist (MQC), [9] a 7-item appraisal tool, was used to assess the quality of each review. Reviews were rated from 0 to 7, with those scoring four or more deemed moderate-to-high quality, and reviews scoring three or fewer deemed poor quality and subsequently excluded. At the time of writing there was no recognised measure for assessing the quality of stakeholder documentation. As such, an adapted version of the MQC, the Methodological Quality Checklist for Stakeholder Documents and Position Papers (MQC-SP), was applied [12]. Stakeholder documentation scoring four or more was considered moderate-to-high quality. Documentation scoring three or fewer was excluded. Quality appraisal was conducted by three reviewers (EB, JI, JBA).

#### Quality of empirical evidence informing review-level evidence

The quality of empirical evidence informing each of the reviews was categorised according to one of three groups: high, moderate, and low. In most cases, allocation of quality rating was based on the rating provided by the original author(s) of each review. However, in some cases a quality rating was not provided by the original authors of a review, and as such, the quality of the empirical evidence could not be determined. In such instances, a quality rating of ‘not reported’ was used.

### Data synthesis

Findings from each review were grouped according to presence of what we defined as a ‘modifiable feature’. This is a feature of the built and natural environment that, if altered in some way, is associated with a positive impact upon people’s behaviours or lifestyles, and/or health outcomes. For example, an improvement in lighting within the home environment (modifiable feature) was found to be associated with improved social outcomes (behavioural impact) and reduced fall-related injuries among older adults (health outcome). Each modifiable feature was then categorised into a broader theme, known as a ‘planning principle’. For example, the modifiable feature ‘improved residential lighting’ was categorised into the broader planning principle ‘improved quality of housing’. In some instances, more than one piece of review-level evidence reporting on the same health impacts and/or outcomes was identified. To avoid possible duplication or over-stating of results, planning principles and modifiable features were generated from the review-level evidence deemed to be of the highest methodological quality. This process was followed for all documentation included in this review, according to each of the five topics of interest.

## Results

**Fig. 1:**
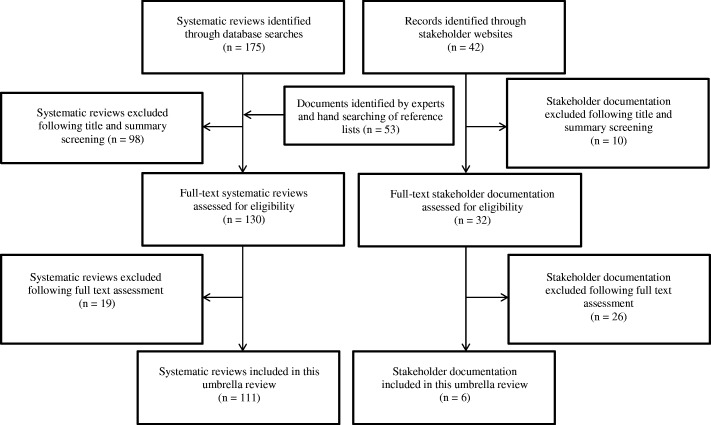
Flowchart for eligible systematic reviews and stakeholder documents

**Fig. 2:**
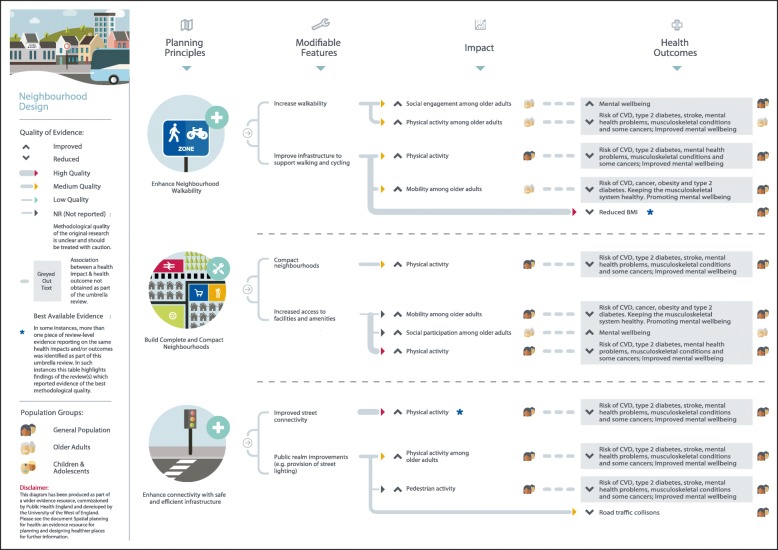
Neighbourhood design planning principles and modifiable features

**Fig. 3:**
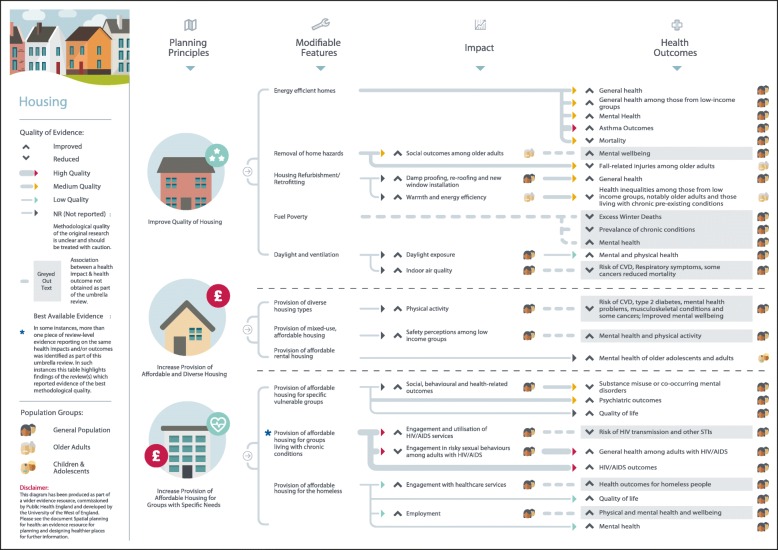
Housing planning principles and modifiable features

**Fig. 4:**
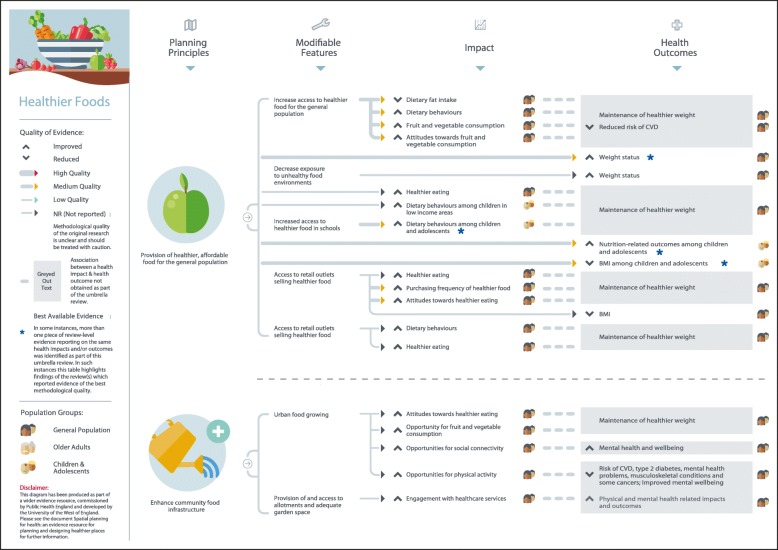
Healthier food environment planning principles and modifiable features

**Fig. 5:**
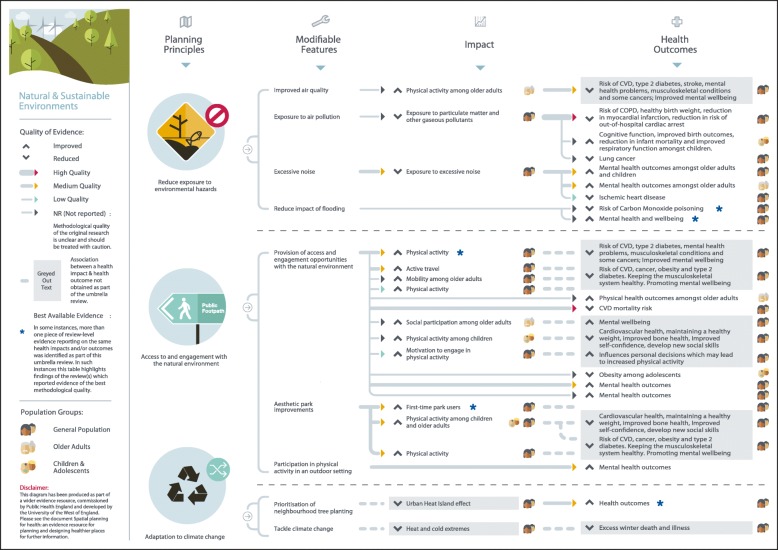
Natural and sustainable environment planning principles and modifiable features

**Fig. 6:**
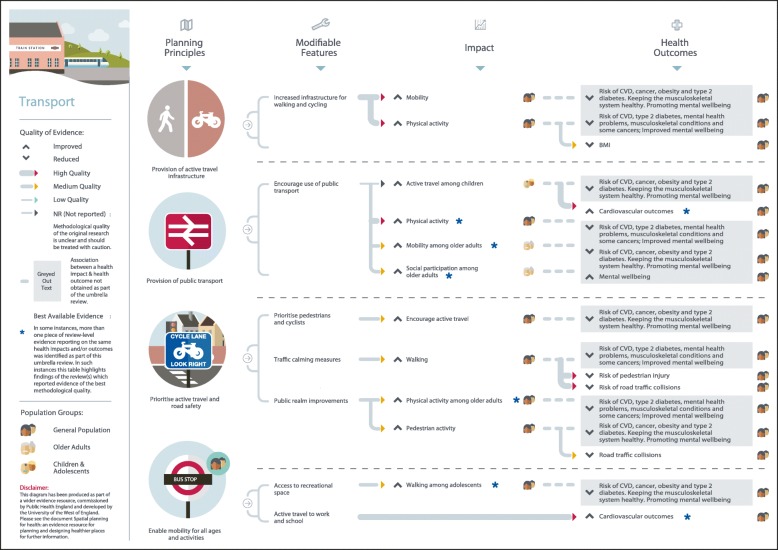
Transport planning principles and modifiable features

Figure 1 summarises the search results. A total of 117 systematic reviews and review-level documents met the inclusion criteria. Many reviews addressed more than one of the five built and natural environment categories suggesting that although there are distinctions between each of the areas, they are also strongly interconnected. In such instances, findings relevant to each topic area were extracted separately and this resulted in the following breakdown of eligible reviews for each category: neighbourhood design (*N* = 32), housing (*N* = 23), healthier food environment (*N* = 20), natural and sustainable environment (*N* = 49), and, transport (*N* = 29). A full reference list of included studies, details of study characteristics and quality can be found in the online additional material. Figures 2 to 6 present a visual representation of findings for each of the built and natural environment topics of interest.

Eligible reviews were based on empirical evidence utilising a range of study designs and were targeted at a variety population groups. The quality of evidence was mixed as much of the evidence examined relied on findings from cross-sectional studies, making it difficult to draw clear causal links between specific built and natural environment principles and features and health-related outcomes: one review included original empirical studies deemed to be high quality (1%), 11 reviews contained evidence of moderate-to-high quality (9%), 25 of moderate quality (25%), 14 of low-to-moderate quality (12%), 9 of low quality (8%), and for 52 reviews the quality of evidence from original empirical studies was not reported by review authors (45%). In considering the evidence presented here, policy makers should be mindful that an absence of high quality evidence does not mean an association between a planning principle and health outcome does not exist. Further, many planning interventions can be expected to confer co-benefits either to both health and other outcomes, or across a number of health outcomes. Only four reviews eligible for inclusion focused on health inequalities; some positive results were reported, but the overall picture on associations between the built environment and health inequalities was inconclusive. The planning principles, modifiable features, and health-related outcomes identified for each built and natural environment domain, alongside an assessment of evidence quality, are summarised in the text below and in Table 2.

**Table 2:** Built and natural environment planning principles and modifiable features associated with improved health-related outcomes

Neighbourhood Design	
*Enhance neighbourhood walkability*	
Increase walkability	Increased social engagement S9 (2); S15 (N/R); S16 (1–2)
	Increased mobility S9 (2); S15 (N/R); S16 (1–2)
	Increased physical activity S1 (2); S2 (3); S3 (1–2); S4 (2); S5 (2–3); S6 (N/R); S7 (2); S8 (2); S9 (2); S10 (N/R); S11 (N/R); S12 (N/R); S13 (1); S14 (N/R); S15 (N/R)
Improve infrastructure to support walking and cycling	Increased physical activity S1 (2); S2 (3); S3 (1–2); S4 (2); S5 (2–3); S6 (N/R); S7 (2); S8 (2); S9 (2); S10 (N/R); S11 (N/R); S12 (N/R); S13 (1); S14 (N/R); S15 (N/R)
	Increased mobility among older adults S9 (2); S15 (N/R); S16 (1–2)
	Improved weight status S17 (N/R); S18 (N/R); S19 (2)
*Build complete and compact neighbourhoods*	
Compact neighbourhoods	Increased physical activity S4 (2); S9 (2); S14 (N/R); S21 (1–2); S23 (2)
Increase access to facilities and amenities	Increased mobility among older adults S4 (2); S9 (2); S14 (N/R); S21 (1–2); S23 (2)
	Increased social participation among older adults S27 (N/R)
	Improved mental health S24 (1–2); S25 (1–2); S26 (2–3)
*Enhance connectivity with safe and efficient infrastructure*	
Improved street connectivity	Increased physical activity S1 (2); S2 (3); S3 (1–2); S4 (2); S5 (2–3); S6 (N/R); S7 (2); S8 (2); S9 (2); S10 (N/R); S11 (N/R); S12 (N/R); S13 (1); S14 (N/R); S15 (N/R)
Provision of public realm improvements (e.g. provision of street lighting)	Increased physical activity S6 (N/R); S9 (2); S14 (N/R); S112 (1–2)
	Reduced fear of crime S28 (1)
	Reduced road traffic collisions S29 (1); S112 (1–2); S113 (2–3)
Housing	
*Improve quality of housing*	
Increase energy efficient homes	Improved general and mental health outcomes (including for those from low-income groups) S15 (N/R); S25 (1–2); S32 (2); S33 (N/R); S35 (2–3); S36 (2); S42 (N/R)
	Reduced mortality S31 (1–2)
Remove home hazards	Improved social outcomes among older adults S39 (2); S40 (2); S42 (N/R)
	Reduced fall-related injuries among older adults S39 (2); S40 (2)
	Reduced unintentional injury S41 (1–2); S42 (N/R)
Home refurbishment/retrofit	Improved general health S25 (1–2); S32 (2); S33 (N/R); S34 (N/R); S36 (2)
	Reduced fear of crime S28 (1)
*Increase provision of affordable and diverse housing*	
Provision of diverse housing	Increased physical activity S21 (1–2)
Provision of mixed-use, affordable housing	Increased perceptions of safety among low-income groups S39 (2)
Provision of affordable rental housing	Improved mental health among adolescents and adults S25 (1–2); S32 (2)
*Increase provision of affordable housing for groups with specific needs*	
Provision of affordable housing for vulnerable groups	Improved social outcomes S48 (1)
	Improved behavioural outcomes S45 (2); S51 (2)
	Improved health-related outcomes S48 (1); S50 (N/R)
	Reduced in substance misuse or co-occurring mental disorders S45 (2); S51 (2)
	Improve psychiatric health outcomes S45 (2); S51 (2)
	Increased quality of life S45 (2); S51 (2)
Provision of affordable housing for groups living with chronic conditions	Increased engagement with HIV/AIDS services S43 (2–3); S45 (2); S47 (2)
	Reduced engagement in risky sexual behaviours among those with HIV/AIDS S43 (2–3); S45 (2); S47 (2)
	Improved HIV/AIDS outcomes S43 (2–3)
Provision of affordable housing for the homeless	Increased engagement with healthcare services S46 (2); S49 (N/R)
	Increased quality of life S46 (2); S49 (N/R)
	Increased employment S44 (1)
	Improved mental health S46 (2); S49 (N/R)
Healthier Food Environment	
*Increase provision of healthier, affordable food*	
Increase access to healthier food	Reduced dietary fat intake S52 (N/R)
	Improved dietary behaviour S52 (N/R); S53 (1–2); S54 (1); S55 (2); S56 (2–3); S57 (2); S58 (N/R)
	Increased fruit and vegetable intake S39 (2); S52 (N/R)
	Improved attitudes towards fruit and vegetables S39 (2); S52 (N/R)
	Improved weight status S61 (N/R); S62 (2); S63 (1)
	Healthier food purchasing S59 (N/R); S60 (2)
*Enhance community food infrastructure*	
Increase urban food growing	Improved attitudes towards healthier eating S64 (N/R)
	Increased opportunities for fruit and vegetable consumption S9 (2); S64 (N/R)
	Increased opportunities for social connectivity S9 (2); S64 (N/R)
	Increased opportunities for physical activity S9 (2); S64 (N/R)
Natural and Sustainable Environment	
*Reduce exposure to environmental hazards*	
Improve air quality	Increased physical activity among older adults S24 (1–2)
Reduce exposure to air pollution	Reduced risk of chronic conditions S70 (2); S71 (N/R); S72 (2); S73 (N/R); S74 (1–2); S75 (1–2); S76 (N/R); S77 (N/R); S78 (N/R); S79 (N/R); S80 (2); S81 (N/R); S82 (N/R); S83 (N/R); S84 (2–3); S85 (N/R); S86 (N/R); S87 (N/R); S88 (2–3); S89 (N/R); S90 (N/R); S91 (N/R); S92 (2–3); S93 (1–2); S94 (N/R)
	Improved birth outcomes S15 (N/R); S95 (N/R); S96 (2); S97 (N/R); S98 (N/R); S99 (N/R)
	Reduced infant mortality S98 (N/R)
	Improved cognitive function S100 (N/R)
Reduce exposure to excessive noise	Improved mental health outcomes S15 (N/R); S24 (1–2); S25 (1–2)
	Reduced risk of ischemic heart disease S101 (N/R)
Reduce impact of flooding	Reduced risk of carbon monoxide poisoning S102 (N/R)
	Improved mental and physical health outcomes S103 (N/R); S104 (N/R); S105 (N/R)
*Increase access to, and engagement with, the natural environment*	
Increase access and engagement opportunities	Increased physical activity S9 (2); S21 (N/R); S111 (N/R); S107 (1); S117 (N/R)
	Reduced risk of cardiovascular disease S24 (1–2); S26 (2–3)
	Increased motivation to engage in physical activity S108 (1); S117 (N/R)
	Reduced obesity among adolescents S18 (N/R); S111 (N/R)
	Improved mental health outcomes S24 (1–2); S26 (2–3); S109 (2)
Aesthetic park improvements	Increased first-time park users S9 (2); S107 (1)
	Increased physical activity S9 (2); S18 (N/R); S107 (1); S111 (N/R)
*Adaptation to climate change*	
Prioritisation of neighbourhood tree planting	Improved health outcomes S110 (2)
Transport	
*Provision of active travel infrastructure*	
Increase infrastructure for walking and cycling	Increased physical activity S1 (2); S2 (3); S3 (1–2); S4 (2); S5 (2–3); S6 (N/R); S7 (2); S8 (2); S9 (2); S10 (N/R); S11 (N/R); S12 (N/R); S13 (1); S14 (N/R); S15 (N/R)
	Increased mobility among S9 (2); S15 (N/R); S16 (1–2)
	Improved weight status S17 (N/R); S18 (N/R); S19 (2)
*Enhance connectivity with safe and efficient infrastructure*	
Provision of traffic calming measures	Increased physical activity S6 (N/R); S9 (2); S112 (1–2); S14 (N/R)
	Reduced risk of pedestrian injury S29 (1); S112 (1–2); S113 (2–3)
	Reduced risk of road traffic collision S29 (1); S112 (1–2); S113 (2–3); S114 (2); S115 (N/R); S116 (1–2)
	Increased pedestrian activity S112 (1–2)
Provision of public realm improvements (e.g. provision of street lighting)	Increased physical activity S6 (N/R); S9 (2); S14 (N/R); S24 (1–2); S112 (1–2)
	Reduced fear of crime S28 (1)
	Reduced road traffic collisions S29 (1); S112 (1–2); S113 (2–3)
*Prioritise public transport*	
Promote public transport use	Increased physical activity S111 (N/R)
	Improved cardiovascular outcomes S19 (2)
	Reduced fear of social isolation S15 (N/R)
	Improved mental health S15 (N/R)
*Enable mobility for all ages and activities*	
Increase access to recreational space	Improved pedestrian safety among adolescents S112 (1–2)
	Improved mental health S25 (1–2); S26 (2–3)

Note. S1–117 = Review-level evidence included in this review. See Additional files 1, 2 and 3 for full reference list. () = Quality of original empirical studies included within review-level evidence, as assigned by review authors. 1 = Low quality, 2 = Moderate quality, 3 = High quality, N/R = Not reported by authors of review

### Neighbourhood design

As shown in Fig. 2, three planning principles were identified through this umbrella review: enhance neighbourhood walkability; build complete and compact neighbourhoods; and, enhance connectivity with safe and efficient infrastructure. Neighbourhoods with features including street connectivity, mixed land use and compact residential design, were found to be associated with higher or increased physical activity among the general population,[S1-S15] and higher or increased social engagement and mobility among older adults [S9, S15-S16]. Moderate-to-high quality reviews reported a positive or null association [S17-S19] between infrastructure for walking and cycling and weight status [S20]. Evidence of mixed methodological quality suggested that densely populated neighbourhoods with good access to local facilities and amenities were associated with higher or increased physical activity and mobility,[S9, S14, S21, S23] higher or improved mental health,[S24-S26] and higher social participation among older adults [S27]. Improvements to safety and efficiency of neighbourhood infrastructure, for example provision of quality street lighting, was associated with higher physical activity,[S9] and lower fear of crime [S28] and road traffic collisions [S29, S112-S113]. One review examining the effectiveness of built environment interventions in managing symptoms of dementia reported lower behavioural symptoms following the redesign of existing physical space [S30].

### Housing

Improvements in housing quality, such as increased energy efficiency, were found to be associated with positive general health, mental health, asthma, and mortality outcomes [S15, S25, S31-S38] (Fig. 3). Improvements in warmth energy efficiency were also found to be associated with reduced health inequalities among older adults and those with chronic conditions from low-income groups [S32]. Moderate quality evidence indicates that lighting improvements were associated with increased or higher social engagement and reduced fall-related injuries,[S39-S40] however, one review reported that the impact of such an intervention on reducing health inequalities was unclear [S39]. Home safety measures such as smoke alarm installation and pre-set safe temperature hot water heaters were associated with a reduction in, or lower rates of, unintentional injury [S41-S42]. The provision of affordable and diverse housing was found to be associated with higher or increased physical activity, primarily walking [S21] and perceived safety among those from low income groups [S39]. Affordable rental housing, specifically, was associated with higher or improved mental health outcomes among adolescents and adults [S25, S32]. The provision of affordable housing to vulnerable individuals with specific needs, including those living with intellectual disability, substance users, homeless, and those living with a chronic condition was associated higher or improved social, behavioural, physical and mental health-related outcomes [S43-S51].

### Food environment

Good quality evidence on the associations between the food environment and health outcomes is relatively sparse, and what has been published is contradictory in places (Fig. 4). Evidence of moderate or unreported methodological quality indicates that the provision of healthier, affordable food in specific delivery settings (e.g., schools, workplaces, supermarkets) is associated with higher or improved dietary behaviours,[S52-S58] higher or improved attitudes towards fruit and vegetable consumption,[S39, S52] healthier food purchasing,[S59-S60] and positive associations with weight-related health outcomes [S61-S63]. However, one review found no evidence for an associations between affordable food and energy, fat, or sugar intake,[S55] and another found no association with weight-related outcomes [S52]. Enhancing community food infrastructure through urban food growing and provision of and access to allotments and garden space was related to positive attitudes towards healthier eating,[S64] higher opportunities for fruit and vegetable consumption, social connectivity, physical activity and engagement with healthcare [S9, S64]. Other reviews reported mixed, inconclusive findings for associations between the food environment and health-related outcomes[S22, S65–69]. Overall, it is important to exercise caution when interpreting these findings as review-level evidence draws upon empirical evidence that is cross-sectional in nature, restricting our ability to draw causal links.

### Natural and sustainable environment

This review identified a wealth of mixed-quality review-level evidence linking the natural and sustainable environment with health. Reduced exposure to environmental hazards, such as poor air quality, was associated with increased physical activity among older adults[S24] (Fig. 5). Exposure to air pollution was related to a higher or an increased risk of chronic conditions,[S70-S94] worsened birth outcomes,[S15, S95-S99] and problems with cognitive function [S100]. Exposure to excessive noise was linked to lower mental health outcomes,[S15, S24-S25] and higher risk of ischemic heart disease[S101]. Available evidence on the health risks of flooding suggest a higher risk of carbon monoxide poisoning [S102] and adverse long-term impacts on mental health[S103-S105]. Access to, and engagement with, the natural environment was associated with numerous positive physical and mental health outcomes[S9, S18, S21, S24-S26, S107-S109, S111, S117]. Moderate quality evidence from one review revealed that neighbourhood tree planting (also known as ‘greening’) was associated with higher health outcomes[S110].

### Transport

As shown in Fig. 6, four planning principles associating transport with a range of health impacts and outcomes were identified. Moderate-to-high quality evidence suggests that provision of active travel infrastructure for walking and cycling is associated with higher or increased mobility and physical activity [S1-S15]. Four moderate-to-high quality reviews reported a positive or null association [S17-S19] between infrastructure for walking and cycling and weight status [S20]. Provision of public transport was found to be associated with higher physical activity [S111], better cardiovascular outcomes in the general population,[S19] and a lower fear of social isolation and improved mental health[S15]. Initiatives to prioritise active travel and road safety, such as traffic calming measures, were associated with a range of positive physical activity behaviours,[S6, S9, S14, S112] a lower or reduced risk of road traffic collisions and pedestrian injury[S29, S112-S116] and a lower fear of crime [S28]. Moderate neighbourhood tree planting (alsoquality evidence revealed that enabling mobility for all ages and activities through increased access to aesthetically pleasing recreational space was associated with positive mental health outcomes [S25-S26]. It has also been shown to be associated with better pedestrian safety and higher walking among adolescents, although the evidence was less clear among children [S112].

## Discussion

This umbrella review provides an up-to-date overview of the evidence for associations between the built and natural environment and health. The review identifies fourteen evidence-based, actionable planning principles related to five distinct, yet interconnected aspects of the built and natural environment: neighbourhood design, housing, food environment, natural and sustainable environment, and transport. In accordance with previous research, [2] evidence for the effectiveness of planning principles on reducing health inequalities was sparse and inconclusive. Overall, the findings of this review build upon previous research in this area, [2, 9, 10, 12] while also contributing a novel practical approach to guide the planning and development of future built and natural environment interventions and policies.

### Strengths and limitations

A key strength of this study is the robustness and rigour of the umbrella review methods applied. Evidence reviews deemed to be of low quality were excluded from the final analysis and findings are therefore based on the best available and current evidence. However, caution is advised as much of the review-level evidence examined was reliant on findings from cross-sectional studies. In a complex system [4] such as the built and natural environment, it is rarely possible or appropriate to undertake an experimental approach (such as a randomised controlled trial) that can in other circumstances offer the best way of assessing causality. Wherever possible, experimental approaches should be undertaken in order to develop the evidence base in this field, as they do often offer the highest quality of evidence regarding causality. The findings of this review are also limited in their inability to draw firm conclusions about the impact of the built and natural environment on health inequalities, as studies with a specific health inequality outcome were extremely rare.

The decision to focus purely on review-level evidence has its drawbacks. Despite an extensive search of the literature it was soon realised that evidence from some original empirical studies has yet to be systematically reviewed. Importantly, this does not mean that characteristics of the built and natural examined to date only in empirical studies are not important. Additional work could broaden the scope of the review to include assessment of individual empirical studies, although it is acknowledged that this would represent a significant undertaking. Finally, the inclusion of qualitative evidence reviews was beyond the remit of this umbrella review, but future large-scale reviews may benefit from the inclusion of qualitative evidence to explore the relationship between health and the built and natural environment from a more in-depth perspective.

### Implications for policymakers

Findings from this review strengthen the argument for an upstream shift to address key built and natural environment obstacles to enable people to increase control over, and improve, their health. Communication between built environment and health professionals is essential. Findings highlight the importance of local evidence-based action to ensure settings- and place-based approaches provide opportunities for people to live healthier lives. Incorporating health needs and impact into the conceptualisation, design and planning of infrastructural projects, may assist policy makers, planners and built environment professionals in the development of sustainable communities.

Findings of this umbrella review were used to produce a series of diagrams to assist public health and planning professionals in designing places that enhance the health and wellbeing of local people. The diagrams clearly show where the evidence-based links exist between planning principles and health outcomes, indicating the strength of the evidence, and the population groups that have been shown to benefit. Although presented as five separate aspects of the built and natural environment, the evidence indicates interconnection between areas, particularly in terms of actionable planning principles and modifiable features. As such, we recommend that the results from this umbrella review, and supporting diagrams for each topic area, are taken together to provide a broad overview of the evidence and to encourage thinking that extends beyond a silo mentality. It is hoped these resources will provide a useful tool to promote better engagement between public health and planning professionals, so that health can be effectively designed in to spatial planning developments.

## Conclusions

This umbrella review provides an up-to-date overview of the evidence for associations between the built and natural environment and health. The review identifies evidence-based, actionable planning principles, related to five distinct aspects of the built and natural environment, and contributes a novel practical approach to guide the planning and development of future built and natural environment interventions and policies.

### What is already known on this subject

- The natural and built environment plays a key role in shaping the social and economic determinants of health.
- Although associations between the environment and health have long been established, there is often insufficient evidence to ascertain causality.

### What this study adds

- This study systematically assessed evidence from recent systematic reviews on the association between the built and natural environment and health. The collation of evidence provides readers with an overview of the research that has been conducted in this field.
- The findings demonstrate evidence-based links between planning principles and health outcomes to aid communication among planners and public health professionals.

## Additional files

Additional file 1: List of stakeholder organisation websites searched (alphabetical order). This file lists the stakeholder organisation websites searched for eligible review level documentation. (DOCX 14 kb)

Additional file 2: Characteristics of eligible review-level evidence, according to domain of interest. This file details the characteristics of eligible review-level evidence included within the umbrella review, and includes study population characteristics and quality appraisal outcomes. (DOCX 47 kb)

Additional file 3: Reference list of eligible review-level evidence. This file contains the full reference list for eligible review-level documentation included within this umbrella review. (DOCX 43 kb)

## Abbreviations

MQC: Methodological Quality Checklist
MQC-SP: Methodological Quality Checklist for Stakeholder Documents and Position Papers
