# Supplementary material for: Built and natural environment planning principles for promoting health: an umbrella review
Source: BMC Public Health. 2018 Jul 28;18:930. doi: 10.1186/s12889-018-5870-2 (PMC6064105; doi:10.1186/s12889-018-5870-2)
Supplement: Supplementary file 1 — List of stakeholder organisation websites searched (alphabetical order). This file lists the stakeholder organisation websites searched for eligible review level documentation. (DOCX 14 kb) [file 12889_2018_5870_MOESM1_ESM.docx]

Additional file 1. List of stakeholder organisation websites searched (alphabetical order)

- Centers for Disease Control and Prevention (CDC)
- Department for Transport (DfT)
- Design Council (which incorporates the work of CABE)
- European Commission (EC)
- HACT Ideas and innovations in housing
- Landscape Institute
- National Institute for Health and Care Excellence (NICE)
- Public Health England (PHE)
- Royal Institute of British Architects (RIBA)
- Royal Society for the Prevention of Accidents (ROSPA)
- Royal Town Planning Institute (RTPI)
- Spatial Planning and Health Group (SPAHG)
- Town and Country Planning Association (TCPA)
- UK Health Forum
- World Health Organisation (WHO)
