# Supplementary material for: Built and natural environment planning principles for promoting health: an umbrella review
Source: BMC Public Health. 2018 Jul 28;18:930. doi: 10.1186/s12889-018-5870-2 (PMC6064105; doi:10.1186/s12889-018-5870-2)
Supplement: Supplementary file 2 — Characteristics of eligible review-level evidence, according to domain of interest. This file details the characteristics of eligible review-level evidence included within the umbrella review, and includes study population characteristics and quality appraisal outcomes. (DOCX 47 kb) [file 12889_2018_5870_MOESM2_ESM.docx]

Additional file 2. Characteristics of eligible review-level evidence, according to domain of interest

| **First author, year of publication** | **Original studies^a^** | **Design of original studies^b^** | **Population** | **Location^c^** | **Outcome of interest** | **Review quality^d^** | **Evidence quality^e^** |
| --- | --- | --- | --- | --- | --- | --- | --- |
| **Neighbourhood design** | | | | | | | |
| Annear, 2014 | 83 | C-S, L, RCT | Older adults | HICs | Healthy active ageing | 5 | 1-2 |
| Beard, 2010 | 51 | C-S, L | Older adults | N/R | Active ageing | 5 | 1-2 |
| Beyer, 2009 | 17 | CBA | General population | USA, UK, Australia, Germany | Road traffic injuries | 6 | 1 |
| Cairns, 2014 | 5 | S-R | General population | UK | General health and inequalities | 7 | 2-3 |
| Carlin, 2015 | 12 | RCT, Q | Age 5-18 years | UK, USA, Australia, Taiwan | Physical activity (walking) | 7 | 2 |
| Casey, 2014 | 25 | C-S, L | Boys and girls | USA, Australia | Childhood obesity | 5 | N/R |
| Clark, 2007 | 99 | C-S | General population | HICs | Physical and mental health | 5 | 1-2 |
| D’Haese, 2015 | 68 | C-S, C-C | Age 6-12 years | North America, Europe | Physical activity (active travel) | 7 | 3 |
| Dunton, 2009 | 15 | C-S | Children and Adolescents | USA, Canada | Childhood obesity | 4 | N/R |
| Durand, 2011 | 44 | C-S, L, quasi-L | General population | HICs | Physical activity, obesity risk | 5 | 1-2 |
| Faulkner, 2009 | 13 | C-S | Children | USA, Philippines, Australia, UK, Denmark, New Zealand | Physical activity, body weight | 5 | N/R |
| Fraser, 2011 | 21 | C-S, L, | General population | HICs | Physical activity (cycling) | 6 | 1-2 |
| Gascon, 2015 | 12 | Eco, C, CS | General population | UK, US, Oceania, Canada, Lithuania | Mental health | 7 | 2-3 |
| Gomez, 2015 | 8 | C-S, L, C-C, RCT, CR-CS | General population | HICs, Latin America | Physical activity | 6 | 2 |
| Grasser, 2013 | 34 | N/R | Adults | N/R | Physical activity and weight-related outcomes | 6 | 2-3 |
| Hajna, 2015 | 6 | unclear | Adults | Europe, Asia | Physical activity (walking) | 4 | N/R |
| Larouche, 2014 | 73 | C-S, RCT, C-C | N/R | UK, USA, Sweden, Denmark, Portugal, Philippines | Physical activity, body composition and cardiovascular fitness | 7 | 2 |
| Leavsseur, 2015 | 50 | C-S, Qual. | Older adults | HICs | Mobility and social isolation | 5 | N/R |
| Lorenc, 2013 | 47 | CBA, uBA | General population | UK, USA, Netherlands | Fear of crime | 7 | 1 |
| Mayne, 2015 | 37 | CS, L | N/R | USA, UK, Australia, Canada, New Zealand, Chile | Obesity | 7 | 2 |
| McCormack 2011 | 33 | C-S, Q | General population | HICs | Physical activity | 5 | 2 |
| Mueller, 2015 | 30 | HIA, CBA | N/R | USA, Netherlands, Spain, UK, Australia, Norway | Physical activity (active travel) | 6 | N/R |
| Rothman, 2014 | 85 | Quantitative | Age 0-12 years | Western Europe, Australia, Japan, North America, New Zealand | Physical activity | 7 | 1-2 |
| Soril, 2014 | 5 | Comparative cohort | Residents with dementia in long-term care settings | Australia, Canada, UK, USA | Dementia | 7 | 1-2 |
| van Cauwenberg, 2011 | 31 | C-S, L | Older adults | North America, Europe, Australia, Asia | Physical activity | 5 | N/R |
| van Holle, 2012 | 70 | C-C | General population | Europe | Physical activity | 5 | N/R |
| Wanner, 2012 | 46 | C-S, | General population | Europe, North America | Physical activity, body weight | 6 | 1 |
| WHO, 2007 | N/R | N/R | General population | Worldwide | Obesity | 5* | N/R |
| WHO, 2012 | N/R | N/R | General population | Worldwide | Health inequalities | 5* | N/R |
| Xu, 2013 | 19 | C-S, S-R,C-S | General population | Unclear | Physical activity, cardiovascular health, body weight | 7 | 2 |
| Yen, 2009 | 33 | C-S, L | Older adults | HICs | General health | 5 | 2 |
| **First author, year of publication** | **Original studies^a^** | **Design of original studies^b^** | **Population** | **Location^c^** | **Outcome of interest** | **Review quality^d^** | **Evidence quality^e^** |
| **Housing** | | | | | | | |
| Aidala, 2016 | 111 | RCT, UC-C, Case-C | Included adults with HIV/AIDS | USA, Canada, France, Spain, Italy, Finland, South Korea | Health outcomes | 7 | 2-3 |
| Bambra, 2010 | 9* | SR | Adults | USA | General health and health inequalities | 7 | 2 |
| Bassuk, 2014 | 6 | UR-CS, T-S | Included homeless families | USA | Homelessness | 7 | 1 |
| Clark, 2007 | 99 | L, C-S | General population | Australasia, Europe, Japan, North America | Physical and mental health | 4 | 1-2 |
| DiGuiseppi, 2011 | N/R | RCT | General population | N/R | Injury prevention | 4 | 1-2 |
| Durand, 2011 | 44 | C-S, L, quasi-L | General population | HICs | Physical activity, obesity risk | 5 | 1-2 |
| Fitzpatrick-Lewis, 2011 | 10 | RCT, Q, UC-C, C-S | Included homeless, marginally housed, or at risk of homelessness | N/R | Homelessness | 7 | 2 |
| Garin, 2014 | 48 | L, CS | Older adults | HMICs | General health | 5 | 1-2 |
| Gibson, 2011 | 5* | SR | Disadvantaged groups | US, UK, New Zealand, Europe | Health inequalities | 6 | 2 |
| Krieger, 2014 | N/R | RCT | Asthmatics | N/R | Asthma | 4 | N/R |
| Kyle, 2008 | 29 | RCT, Q UC-C, C-S | Adults with severe and persistent mental illness | USA, Canada, Denmark, Australia, New Zealand, England | Health, quality of life, healthcare use | 6 | 2 |
| Leaver, 2007 | 17 | CR-CS, L, C-S | Included adults with HIV/AIDS | USA, EU, Canada, Australia, Ivory Coast | General health | 7 | 2 |
| Lindberg, 2010 | N/R | RCTs | N/R | N/R | General health | 4 | N/R |
| Lorenc, 2013 | 47 | CBA, uBA | General population | UK, USA, Netherlands | Fear of crime | 7 | 1 |
| Mansell, 2009 | 19 | C-S, CR-CS | Included adults intellectual disability | UK, Ireland, Netherlands, Australia | General health | 5 | N/R |
| McClure, 2008 | 6 | CBA | Older adults | Australia, Denmark, Sweden, Norway, Taiwan | Fall-related injuries | 7 | 2 |
| Nelson, 2007 | 16 | E, Q | Mentally ill and history of homelessness | US | Homelessness | 5 | N/R |
| Reif, 2014 | 5 | RCT, Q | Substance users or co-occurring mental and substance use disorders | US | General health | 6 | 2 |
| Sandel, 2010 | N/R | N/R | N/R | N/R | Health-related chemical agents | 4 | N/R |
| Thomson, 2009 | 40 | RCT, C-C | N/R | N/R | General health | 7 | 2-3 |
| Thomson, 2013 | 39 | RCT, CR-CS, UR-CS, UBA, CBA | General population | Worldwide | General health | 7 | 2 |
| WHO, 2005 | N/R | C-S, E | General population | HMICs | Obesity | 5* | N/R |
| WHO, 2012 | N/R | N/R | General population | Worldwide | Health inequalities | 5* | N/R |
| **First author, year of publication** | **Original studies^a^** | **Design of original studies^b^** | **Population** | **Location^c^** | **Outcome of interest** | **Review quality^d^** | **Evidence quality^e^** |
| **Healthier food environment** | | | | | | | |
| Bambra, 2010 | 1* | SR | Adults | USA | General health and health inequalities | 7 | 2 |
| Calancie, 2015 | 29 | UBA, Q, C-S | Rural community | USA, Canada | Obesity | 6 | N/R |
| Caspi, 2012 | 38 | C-S, N, CR-CS | General population | USA, Scotland, Australia, UK, Japan, Canada, NZ | Diet | 4 | 2 |
| Cobb, 2015 | 71 | L, C-S | General population | USA, Canada | Obesity | 7 | 1 |
| Delgado-Noguera, 2011 | 3 | RCT, Q | Children | Norway, UK | Fruit and vegetable consumption | 7 | 2 |
| De Vet, 2010 | 7 | SR | Children and adolescents | N/R | Physical activity, diet | 6 | 2-3 |
| Dreissen, 2014 | 18 | RCT, UC-C, C-S | Children | USA, UK | Body weight | 7 | 1-2 |
| Feng, 2010 | 22 | N/R | General population | USA, Australia | Obesity | 4 | N/R |
| Gamba, 2015 | 51 | C-S, L | General population | USA | Obesity | 5 | N/R |
| Ganann, 2014 | 13 | RCT, Q, CR-CS, UC-C | School community | USA, South Africa, Netherlands, UK, France | Nutrition | 7 | 1 |
| Giskes, 2010 | 28 | C-S, N | Adults | USA, Australia, New Zealand, Japan, UK, Netherlands | Diet | 6 | N/R |
| Gittlesohn, 2012 | 16 | RCT, C-S, UR-CS, D | Low income adults | USA, Scotland, Australia | Diet, chronic disease | 5 | N/R |
| Jaime, 2009 | 12 | RCT, Q, CR-CS, UR-CS | Children | USA, Denmark, UK, Norway, Belgium, Netherlands, Spain | Diet, obesity | 5 | N/R |
| Kent, 2014 | 138 | N/R | General population | Worldwide | General health and wellbeing | 4 | N/R |
| McCormack, 2011 | 16 | Undefined | General population | USA | Physical activity | 5 | 2 |
| Osei-Assibey, 2012 | 8 | RCT, Q, UR-CS | Children <9 years | USA, Netherlands, Sweden, UK, Germany | Overweight, obesity | 7 | 2 |
| Roy, 2015 | 15 | RCT, UR-CS, Q, CS | Young adults in tertiary education | USA, UK, Belgium, Italy | Dietary behaviour | 7 | 2-3 |
| Sonntag, 2015 | 8 | C-S, D, N/R | Age 3-11 years | USA, Netherlands, UK, Germany, Sweden, Canada, Australia | Obesity, diet | 6 | 2 |
| Wall, 2006 | 4 | RCT | Adults | Worldwide | Diet | 6 | 2 |
| Williams, 2014 | 30 | L, C-S | Age 5-18 years | USA, Canada, Europe, Australia, Asia | Obesity | 7 | 1 |
| **First author, year of publication** | **Original studies^a^** | **Design of original studies^b^** | **Population** | **Location^c^** | **Outcome of interest** | **Review quality^d^** | **Evidence quality^e^** |
| **Natural and sustainable environment** | | | | | | | |
| Ahern, 2005 | 212 | Epidemiological | General population | Worldwide | General health | 6 | N/R |
| Alderman, 2012 | 35 | Epidemiological | General population | Worldwide | General health | 5 | N/R |
| Annear, 2014 | 83 | RCT, C-S, C | Older adults | Canada, USA, Western Europe | Healthy active ageing | 7 | 1-2 |
| Atkinson, 2014 | 110 | T-S | General population | Global | Mortality, hospital admissions | 5 | 2 |
| Balti, 2014 | 10 | C | General population | USA, Canada, Denmark, Netherlands, Switzerland, Germany | Type II diabetes | 5 | N/R |
| Bonzini, 2010 | 18 | C-C, T-S | Pregnant women | Brazil, Canada, USA, Australia | Birth outcomes | 5 | N/R |
| Boothe, 2014 | 9 | Case-C, and unclear | Children | HICs | Childhood leukaemia | 6 | 2 |
| Bowler, 2010 | 25 | C-O, Q | General population | UK | General health | 6 | 2 |
| CABE, 2009 | N/R | N/R | N/R | N/R | General health and wellbeing | 4* | N/R |
| Calogiuri, 2014 | 90 | SR, Q, C, E | General population | N/R | Physical activity | 4 | N/R |
| Chen, 2014 | 17 | C, Case-C | General population | USA, UK, Italy, Australia, Israel, South Korea, Spain, Taiwan | Congenital abnormalities | 5 | 1-2 |
| Clark, 2007 | 99 | L, C-S | General population | Australasia, Europe, Japan, North America | Physical and mental health | 4 | 1-2 |
| Davison, 2006 | 33 | C-S, L | Age 3-18 years | USA, UK, Portugal, Canada, Australia, | Physical activity | 5 | N/R |
| Deng, 2016 | 10 | L, C-S, Eco | Adult males | USA, Czech Republic, Italy, China, Poland, Turkey | Sperm quality | 6 | 1-2 |
| Dunton, 2009 | 15 | C-S | Children and Adolescents | USA, Canada | Childhood obesity | 4 | N/R |
| Eze, 2015 | 7 | L, C-S | General population | USA, Canada, Germany, Denmark, Netherlands, Sweden | Diabetes mellitus | 6 | N/R |
| Favarato, 2014 | 20 | N/R | Children | USA, Spain, Netherlands, Sweden, S Korea, Germany, China, France, Czech Republic | Asthma | 4 | N/R |
| Frutos, 2015 | 7 | C | Women | N/R | Fertility | 5 | 2 |
| Gascon, 2015 | 28 | L, Eco, CS | General population | UK, Netherlands, US, Oceania | Mental health | 7 | 2-3 |
| Gascon, 2016 | 12 | Eco, C, CS | General population | UK, US, Oceania, Canada, Lithuania | Mortality | 7 | 2-3 |
| Hamra, 2014 | 18 | C, Case-C | N/R | USA, Canada, UK, Italy, Germany, China, Japan, New Zealand | Lung cancer | 5 | N/R |
| Hamra, 2015 | 20 | C, Case-C | N/R | USA, Canada, Sweden, Norway, France, Netherlands, UK, Italy, Germany, Denmark, China, Japan | Lung cancer | 5 | N/R |
| Hu, 2014 | 10 | C, Case-C | Pregnant women | USA, Spain, Sweden, Australia, Netherlands | Hypertensive disorders | 6 | N/R |
| Hunter, 2014 | 12 | Q-11, RCT-1 | General population | USA, Australia | Physical activity | 6 | 1 |
| Jafta, 2015 | 8 | Case-C, C-S | Age 15 years or less | India, South Africa, Spain | Childhood tuberculosis | 7 | 2 |
| Janghorbani, 2014 | 17 | C-C (6), C-S (2), C-CR (6) | General population | USA, Europe, Asia, Canada | Risk of diabetes | 5 | N/R |
| Lacasana, 2005 | 31 | T-S, C-C | Early childhood, pregnant women | UK, Czech Republic, USA, Sweden, Taiwan, Brazil, Mexico, South Korea | Prenatal and childhood health effects | 4 | N/R |
| Lee, 2010 | 37 | CS, Eco, Qual, SR | General population | Australia, USA, UK, Netherlands, Canada, New Zealand, Denmark, Japan | General health | 6 | 1 |
| Lui, 2015 | 11 | C-S (9), L-1 | General population | USA, UK, Netherlands, Europe, Taiwan. Canada | Carotid intima-media thickness | 5 | N/R |
| Luo, 2015 | 31 | T-S, C-CR | General population | USA, Asia, Australasia, South America, Europe | Risk of myocardial infarction | 5 | 2-3 |
| McCormack 2011 | 33 | C-S, Q | General population | HICs | Physical activity | 5 | 2 |
| Mehta, 2011 | 74 | T-S (31), C-C (4), Case-C (3), C-CR (6), C-S (9) | Children | Europe and North America, Latin America, Asia | Acute lower respiratory infections | 4 | N/R |
| Najafi, 2015 | 11 | C-S, C-C | Male adults | China, USA, UK | Sperm quality | 4 | N/R |
| Pedersen, 2014 | 17 | C-C, Case-C | Pregnant women | USA, Europe, Iran, Japan, Australia | Pregnancy induced hypertensive disorders | 4 | N/R |
| Peters, 2015 | 8 | C-C | Older adults | USA | Risk of cognitive decline | 6 | N/R |
| Rodriguez-Villamizar, 2015 | 27 | T-S, C-CR, L, C-C-C | Children | Canada | Respiratory health | 7 | 2-3 |
| Scheers, 2015 | 20 | C-C, E-S | General population | Europe, North America, Eastern Asia | Risk of stroke | 6 | N/R |
| Shah, 2015 | 94 | T-S-(69), C-CR (33) | General population | Brazil, Chile Mexico, South Africa, China, Thailand | Stroke | 5 | N/R |
| Song, 2014 | 44 | T-S, C-C, C-S | General population | Europe, China | Chronic objective pulmonary disease | 4 | N/R |
| Stanke, 2012 | 48 | N/R | General population | USA, China, Vietnam, Poland, Sri Lanka, Germany, Korea, UK | Mental health | 4 | N/R |
| Stieb, 2012 | 62 | C-C, Case-C, E-S | Pregnant women | North America, Europe, Asia, Australia, South America | Birth weight | 6 | N/R |
| Teng, 2013 | 8 | C-CR | General population | USA, Denmark, Finland, Australia | Out-of-hospital cardiac arrest | 7 | 2-3 |
| Thompson Coon, 2011 | 11 | RCT, L | General population | USA, Australia, Switzerland | Physical and mental wellbeing | 7 | 2 |
| Turner, 2010 | 17 | C-C | General population | United States, Canada, France, Brazil, Japan, Germany | Childhood leukaemia | 7 | 1-2 |
| Vienneau, 2015 | 10 | Case-C, Cohort, | General population | Europe | Ischemic health disease | 5 | N/R |
| Vrijheid, 2011 | 10 | Case-c, T-S | Pregnant women | USA, UK | Congenital abnormalities | 5 | N/R |
| Waite, 2014 | 23 | N/R | General population | USA | Carbon monoxide poisoning | 4 | N/R |
| WHO, 2007 | N/R | C-S, E | General population | HMICs | Obesity | 5* | N/R |
| WHO, 2012 | N/R | N/R | General population | Worldwide | Health inequalities | 5* | N/R |
| **First author, year of publication** | **Original studies^a^** | **Design of original studies^b^** | **Population** | **Location^c^** | **Outcome of interest** | **Review quality^d^** | **Evidence quality^e^** |
| **Transport** | | | | | | | |
| Aeron-Thomas, 2005 | 10 | cBA | General population | Australia, Singapore, USA | Road traffic collisions | 7 | 2 |
| Annear, 2014 | 83 | C-S, L, RCT | General population | America, Europe, Australia, Asia | Healthy active ageing | 6 | 1-2 |
| Beard, 2010 | 51 | C-S, L | Older adults | N/R | Active ageing | 5 | 1-2 |
| Beyer, 2009 | 17 | CBA | General population | USA, UK, Australia, Germany | Road traffic injuries | 6 | 1 |
| Cairns, 2014 | 5 | S-R | General population | UK | General health and inequalities | 7 | 2-3 |
| Carlin, 2015 | 12 | RCT, Q | Age 5-18 years | UK, USA, Australia, Taiwan |  | 7 | 2 |
| Casey, 2014 | 25 | C-S, L | Boys and girls | USA, Australia | Childhood obesity | 5 | N/R |
| Clark, 2007 | 99 | C-S, reviews | General population | HICs | Physical and mental health | 5 | 1-2 |
| D’Haese, 2015 | 68 | C-S, C-C | Age 6-12 years | North America, Europe. | Physical activity (active travel) | 7 | 3 |
| Davison, 2006 | 33 | C-S | Age 3-18 years | USA, UK, Portugal, Canada, Australia, | Physical activity | 5 | N/R |
| Dunton, 2009 | 15 | C-S | Children and Adolescents | USA, Canada | Childhood obesity | 4 | N/R |
| Faulkner, 2009 | 13 | C-S | Children | USA, Philippines, Australia, UK, Denmark, New Zealand | Physical activity, body weight | 5 | N/R |
| Fraser, 2011 | 21 | C-S, Q | General population | USA, Australia, UK, Canada, Netherlands | Physical activity (cycling) | 7 | 1-2 |
| Gascon, 2015 | 12 | Eco, C, CS | General population | UK, US, Oceania, Canada, Lithuania | Mental health | 7 | 2-3 |
| Gomez, 2015 | 8 | C-S, L, C-C, RCT, CR-CS | General population | HICs, Latin America | Physical activity | 6 | 2 |
| Grasser, 2013 | 34 | N/R | General population | N/R | Physical activity and weight-related outcomes | 6 | 2-3 |
| Hajna, 2015 | 6 | unclear | Adults | Europe, Asia | Physical activity (walking) | 4 | N/R |
| Høye, 2014 | 19 | CBA | General population | UK, Australia, USA, Belgium, Spain, Netherlands, New Zealand, Italy | General health | 5 | N/R |
| Larouche, 2014 | 73 | C-S, RCT, C-C | N/R | UK, USA, Sweden, Denmark, Portugal, Philippines | Physical activity, body composition and cardiovascular fitness | 7 | 2 |
| Lorenc, 2013 | 47 | CBA, uBA | General population | UK, USA, Netherlands | Fear of crime | 7 | 1 |
| Mayne, 2015 | 37 | CS, L | N/R | USA, UK, Australia, Canada, New Zealand, Chile | Obesity | 7 | 2 |
| McCormack 2011 | 33 | C-S, Q | General population | HICs | Physical activity | 5 | 2 |
| Mueller, 2015 | 30 | HIA, CBA | N/R | USA, Netherlands, Spain, UK, Australia, Norway | Physical activity (active travel) | 6 | N/R |
| Pilkington, 2005 | 14 | UBA, CBA | General population | Canada, Norway, UK, New Zealand, Australia | Road traffic collisions | 7 | 1-2 |
| Rothman, 2014 | 85 | Quantitative | Children age 0-12 | Western Europe, Australia, Japan, North America, New Zealand | Physical activity | 7 | 1-2 |
| Wanner, 2012 | 46 | C-S, | General population | Europe, North America | Physical activity, body weight | 6 | 1 |
| WHO, 2007 | N/R | N/R | General population | Worldwide | Obesity | 5* | N/R |
| WHO, 2012 | N/R | N/R | General population | Worldwide | Health inequalities | 5* | N/R |
| Xu, 2013 | 19 | C-S, S-R, C-S | General population | N/R | Physical activity, cardiovascular health, body weight | 7 | 2 |

Note. (a) Number of studies included in original review level evidence. (b) Study designs. RCT = Randomised controlled trial, CR-CS = Controlled repeat cross-sectional, Q = Quasi-experimental, C-C = Controlled-cohort, C-T= Controlled trial; C-S=Cross-sectional, Case-C=case control, uBA= uncontrolled before and after Study, cBA= controlled before and after Study, S-R=Systematic review, HIA= Health Impact Assessment, CBA= Cost Benefit Analysis, Qual = qualitative, B-A = before after. (c) Geographical location of studies included in original review level evidence. (d) Methodological Quality Checklist (MQC) score (0-3 poor-to-moderate quality; 4-7 moderate-to-high quality); * Methodological Quality Checklist for stakeholder Documents and Position Papers (MQC-SP) score (0-3 poor-to-moderate quality; 4-6 moderate-to-high quality). (e) Quality of evidence from studies included in original review level evidence, as reported by umbrella review authors. 1 = high, 2 = moderate, 3 = low, N/R = not reported.
