# Supplementary material for: Built and natural environment planning principles for promoting health: an umbrella review
Source: BMC Public Health. 2018 Jul 28;18:930. doi: 10.1186/s12889-018-5870-2 (PMC6064105; doi:10.1186/s12889-018-5870-2)
Supplement: Supplementary file 3 — Reference list of eligible review-level evidence. This file contains the full reference list for eligible review-level documentation included within this umbrella review. (DOCX 43 kb) [file 12889_2018_5870_MOESM3_ESM.docx]

Additional file 3. Reference list of eligible review-level evidence

|  | Reference |
| --- | --- |
| S1 | Carlin A, Murphy MH, Gallagher AM. Do interventions to increase walking work? A systematic review of interventions in children and adolescents. *Sports Med* 2015;46(5):515-530. |
| S2 | D’Hease S, Vanwollegham G, Hinckson E, De Bourdeauhuij I, Deforche B, Van Dyck D, Cardon G. Cross-continental comparison of the association between the physical environment and active transportation in children: A systematic review. *IJBNPA* 2015;12(145):DOI 10.1186/s12966-015-0308-z. |
| S3 | Fraser SD, Lock K. Cycling for transport and public health: a systematic review of the effect of the environment on cycling. *European Journal of Public Health* [online] 2011;21(6):738-743. |
| S4 | Gomez LF, Sarmiento R, Ordoñez MF, Pardo CF, de Sá TH, Mallarino CH, Miranda JJ, Mosquera J, Parra DC, Reis R. Urban environment interventions linked to the promotion of physical activity: A mixed methods study applied to the urban context of Latin America. *Social Science & Medicine* [online] 2015;131:18-30. |
| S5 | Grasser G, Van Dyck D, Titze S, Stronegger W. Objectively measured walkability and active transport and weight-related outcomes in adults: a systematic review. *International Journal of Public Health* 2013;58(4):615-625. |
| S6 | Hajna S, Ross NA, Brazeau A, Bélisle P, Joseph L, Dasgupta K. Associations between neighbourhood walkability and daily steps in adults: a systematic review and meta-analysis. *BMC Public Health* [online] 2015;15(1):1. |
| S7 | Larouche R, Saunders TJ, Faulkner GEJ, Colley R, Tremblay M. Associations between active school transport and physical activity, body composition, and cardiovascular fitness: a systematic review of 68 studies. *Journal of Physical Activity & Health* 2014;11(1). |
| S8 | Mayne SL, Auchincloss AH, Michael YL. Impact of policy and built environment changes on obesity-related outcomes: a systematic review of naturally occurring experiments. *Obes Rev* 2015;16(5):362-375. |
| S9 | McCormack GR, Shiell A. In search of causality: a systematic review of the relationship between the built environment and physical activity among adults. *IJBNPA* [online] 2011;8(1):125. |
| S10 | Mueller N, Rojas-Rueda D, Cole-Hunter T, de Nazelle A, Dons E, Gerike R, Götschi T, Panis LI, Kahlmeier S, Nieuwenhuijsen M. Health impact assessment of active transportation: a systematic review. *Preventive Medicine* 2015;76:103-114. |
| S11 | Van Cauwenberg J, De Bourdeaudhuij I, De Meester F, Van Dyck D, Salmon J, Calys P, Deforce B. Relationship between the physical environment and physical activity in older adults: a systematic review. *Health Place* 2011;17(2):458-469. |
| S12 | Van Holle V, Deforche V, Van Cauwenberg J, Goubert L, Maes L, Van de Weghe N, De Bourdeaudhuij I. Relationship between the physical environment and different domains of physical activity in European adults: a systematic review. *BMC Public Health* 2012;12:807. |
| S13 | Wanner M, Götschi T, Martin-Diener E, Kahlmeier S, Martin BW. Active transport, physical activity, and body weight in adults: a systematic review. *American Journal of Preventive Medicine* 2012;42(5):493-502. |
| S14 | WHO Europe. Tackling Obesity by Creating Healthy Residential Environments. 2007. Accessed 19 April 2015 from <http://www.euro.who.int/__data/assets/pdf_file/0012/98697/E90593.pdf>. |
| S15 | WHO Collaborating Centre for Healthy Urban Environments. Health inequalities and determinants in the physical urban environment: Evidence briefing. 2012. Accessed 09 August 2016 from <http://www.apho.org.uk/resource/item.aspx?RID=118056>. |
| S16 | Beard JR, Petitot C. Ageing and urbanization: Can cities be designed to foster active ageing? *Public Health Reviews* [online] 2010;32(2):1. |
| S17 | Casey R, Oppert J, Weber C, Charriere H, Salze P, Bardariotti D, Banos A, Fischler C. Determinants of childhood obesity: What can we learn from built environment studies? *Food Quality and Preference* 2014;31:164-172. |
| S18 | Dunton GF, Kaplan J, Wolch J, Jerrett M, Reynolds KD. Physical environmental correlates of childhood obesity: a systematic review. *Obes Rev* 2009;10(4):393-402. |
| S19 | Xu H, Wen LM, Rissel C. The relationships between active transport to work or school and cardiovascular health or body weight: a systematic review. *Asia Pacific Journal of Public Health / Asia-Pacific Academic Consortium for Public Health* 2013;25(4):298-315. |
| S20 | Faulkner GE, Buliung RN, Flora PK, Fusco C. Active school transport, physical activity levels and body weight of children and youth: a systematic review. *Preventive Medicine* 2009;48(1):3-8. |
| S21 | Durand CP, Andalib M, Dunton GF, Wolch J, Pentz MA. A systematic review of built environment factors related to physical activity and obesity risk: implications for smart growth urban planning. *Obesity Reviews* [online] 2011;12(5):e173- e182. |
| S22 | Cobb LK, Appel LJ, Fanco M, Jones-Smith JC, Nur A, Anderson CAM. The relationship of the local food environment with obesity: A systematic review of  methods, study quality and results. *Obesity* 2015;23(7):1331-1344. |
| S23 | Yen IH, Michael YL, Perdue L. Neighborhood environment in studies of health of older adults: a systematic review. *American Journal of Preventive Medicine* 2009;37(5):455-463. |
| S24 | Annear M, Keeling S, Wilkinson T, Cushman G, Gidlow B, Hopkins H. Environmental influences on healthy and active ageing: A systematic review. *Ageing & Society* 2014;34(4):590-622. |
| S25 | Clark C, Myron R, Stansfeld S, Candy B. A systematic review of the evidence on the effect of the built and physical environment on mental health. *Journal of Public Mental Health* [online] 2007;6(2):14-27. |
| S26 | Gascon M, Triguero-Mas M, Martinez D, Dadvand P, Rojas-Rueda D, Plasencia A, Nieuwenhuijsen MJ. Residential green spaces and mortality: A systematic review. *Environmental International* 2016;86:60-67. |
| S27 | Levasseur M, Généreux M, Bruneau J, Vanasse A, Chabot É, Beaulac C, Bédard M. Importance of proximity to resources, social support, transportation and neighborhood security for mobility and social participation in older adults: results from a scoping study. *BMC Public Health* [online] 2015;15(1):1 |
| S28 | Lorenc T, Petticrew M, Whitehead M, Neary D, Clayton S, Wright K, Thomson H, Cummins S, Sowden A, Renton A. Fear of crime and the environment: systematic review of UK qualitative evidence. *BMC Public Health* 2013;3:1. |
| S29 | Beyer FR, Ker K. Street lighting for preventing road traffic injuries. *Cochrane Database of Systematic Reviews* 2009; 1. |
| S30 | Soril LJJ, Leggett LE, Lorenzetti DL, Silius J, Robertson D, Mansell L, Holyroyd-Leduc J, Noseworth TW, Clement FM. Effective use of the built environment to manage behavioural and psychological symptoms of dementia: A systematic review. *PLoS ONE* 2014;https://doi.org/10.1371/journal.pone.0115425. |
| S31 | Garin N, Olaya B, Miret M, Ayuso-Matos JJ, Power M, Bucciarelli P, Haro JM. Built environment and elderly population health: A comprehensive review. *Clinical Practice & Epidemiology in Mental Health* 2014;10:103-115. |
| S32 | Gibson M, Petticrew M, Bambra C, Sowden AJ, Wright KE, Whitehead M. Housing and health inequalities: A synthesis of systematic reviews of interventions aimed at different pathways linking housing and health. *Health & Place* 2011;17:175-184. |
| S33 | Krieger J, Jacobs DE, Ashley PJ, Baeder A, Chew GL, Dearborn D, Hynes HP, Miller JD, Morley R, Rabito F, Zeldin DC. Housing interventions and control of asthma-related indoor biologic agents: A review of the evidence. *J Public Health Management* 2014;16(5):S11-S20. |
| S34 | Sandel M, Baeder A, Bradman A, Hughes J, Mitchell C, Shaunessy R, Takaro TK,  Jacobs DE. Housing interventions and control of health-related chemical agents: A review of the evidence. *J Public Health Management Practice* 2010;16(5):S24-S33. |
| S35 | Thomson H, Thomas S, Sellstrom E, Petticrew M. The health impacts of housing improvement: A systematic review of intervention studies from 1887 to 2007. *AJPH* 2009;99:S681-S692. |
| S36 | Thomson H, Sellstrom TS, Petticrew M. Housing improvements for health and associated socio-economic outcomes. *Cochrane Database of Systematic Reviews* 2013;2. DOI: 0.1002/14651858.CD008657.pub2. |
| S37 | World Health Organization. Is housing improvement a potential health improvement strategy? 2005. Accessed 09 August 2016 from <http://www.euro.who.int/__data/assets/pdf_file/0007/74680/E85725.pdf>. |
| S38 | World Health Organization. WHO guidelines for indoor air quality: selected pollutants. 2010. Accessed 09 August 2016 from <http://www.euro.who.int/__data/assets/pdf_file/0009/128169/e94535.pdf>. |
| S39 | Bambra C, Gibson M, Sowden A, Wright K, Whitehead M, Petticrew M. Tackling the wider social determinants of health and health inequalities: Evidence from systematic reviews. *J Epidemiol Community Health* 2010;64:284-291. |
| S40 | McClure RJ, Turner C, Peel N, Spinks A, Eakin E, Hughes K. Population-based interventions for the prevention of fall-related injuries in older people. *Cochrane Database of Systematic Reviews* 2008;1:DOI: 10.1002/14651858.CD004441.pub2. |
| S41 | DiGuiseppi C, Jacobs DE, Phelan KJ, Mickalide A, Ormandy D. Housing interventions and control of injury-related structural deficiencies: A review of the evidence. *J Public Health Manag Pract* 2010;16(5):S34-S43. |
| S42 | World Health Organization. Is housing improvement a potential health improvement strategy? 2005. Accessed 09 August 2016 from <http://www.euro.who.int/__data/assets/pdf_file/0007/74680/E85725.pdf>. |
| S43 | Aidala AA, Wilson MG, Shubert V, Gogolishvili D, Globerman J, Rueda S, Bozack AK, Caban M, Rourke SB. Housing status, medical care, and health outcomes among people living with HIV/AIDS: A systematic review. *AJPH Research* 2016;106(1):e1-e22. |
| S44 | Bassuk EL, DeCandia CJ, Tsertsvadze A, Richard MK. The effectiveness of housing interventions and housing and service interventions on ending family homelessness: A systematic review. *American Journal of Orthopsychiatry* 2014;84(5):457- 474. |
| S45 | Fitzpatrick-Lewis D, Ganann R, Ciliska S, Kouyoumdjan F, Hwang S. Effectiveness of interventions to improve the health and housing status of homeless people: A rapid systematic review. *BMC Public Health* 2011;11:638. |
| S46 | Kyle T, Dunn JR. Effects of housing circumstances on health, quality of life and healthcare use for people with severe mental illness: A review. *Health and Social Care* 2008;16(1):1-15. |
| S47 | Leaver CA, Burgh G, Dunn JR, Hwang SW. The effects of housing status on health-related outcomes in people living with HIV: A systematic review of the literature. *AIDS Behav* 2007;11:S85-S100. |
| S48 | Lindberg R, Shenassa, ED, Acevedo-Garcia D, Popkin SJ, Villaveces A, Morley RL. Housing interventions at the neighborhood level and health: a review of the evidence. *Journal of Public Health Management and Practice* 2010;16(5):S44-S52. |
| S49 | Nelson G, Aubry T, Lafrance A. A review of the literature on the effectiveness of housing and support, assertive community treatment, and intensive case management interventions for persons with mental illness who have been homeless. *American Journal of Orthopsychiatry* 2007;77(3):350-361. |
| S50 | Mansell J, Beadle-Brown J. Dispersed or clustered housing for adults with  intellectual disability: A systematic review. *Journal of Intellectual & Developmental*  *Disability* 2009;34(4):313-323. |
| S51 | Reif S, George P, Braude L, Dougherty RH, Daniels AS, Ghose SS, Delphin Rittmon, ME. Recovery housing: Assessing the evidence. *Psychiatric Services* 2014;65(3):295-300. |
| S52 | Calancie L, Jilcott Pitts SB, Khan LK, Fleischhacker S, Evenson KR, Schreiner M, Byker C, Owens C, McGuirt J, Bambridge E, Dean W, Johnson D, Kolodinsky J, Pitch E, Pinard C, Quinn E, Whetstone L, Ammerman A. Nutrition-related policy and environmental strategies to prevent obesity in rural communities: A systematic review of the literature: 2002-2013. *Preventing Chronic Disease* 2015;12:e57. |
| S53 | Dreissen CE, Cameron AJ, Thornton LE, Lai SK, Barnett LM. Effect of changes to the school food environment on eating behaviours and/or body weight in children: A systematic review. *Obesity Reviews* 2014;15:968-982. |
| S54 | Ganann R, Fitzpatrick-Lewis D, Cilska D, Peirson LJ, Warren RL, Fieldhouse P, Delgado-Noguera MF, Tort S, Harris SP, Martinez-Zapata MJ, Wolfenden L. Enhancing nutritional environments through access to fruit and vegetables in schools and homes among children and youth: A systematic review. *BMC Research Notes* 2014;7:422. |
| S55 | Osei-Assibey G, Dick S, Macdiarmid J, Semple S, Reilly JJ, Ellaway A, Cowie H, McNeill G. The influence of the food environment on overweight and obesity in young children: A systematic review. *BMJ Open* 2012;2:e001538. |
| S56 | Roy R, Kelly B, Rangan A, Allman-Farinelli M. Food environment interventions to improve the dietary behaviour of young adults in tertiary education settings: A systematic review. *Journal of the Academy of Nutrition and Dietetics* 2015;115(10):1647-1681. |
| S57 | Caspi CE, Sorensen G, Subramanian SV, Kawachi I. The local food environment and diet: A systematic review. *Health Place* 2012;18(5):1172-1187. |
| S58 | Jaime PC, Lock K. Do school based food and nutrition policies improve diet and reduce obesity? *Preventive Medicine* 2009;48:45-53. |
| S59 | Gittlesohn J, Rowan M, Gadhoke P. Interventions in small food stores to change the food environment, improve diet and reduce risk of chronic disease. Pr*eventing Chronic Disease* 2009;9:110-115. |
| S60 | Sonntag D, Schneider S, Mdege N, Ali S, Schmidt B. Beyond food promotion: A systematic review on the influence of the food industry on obesity-related dietary behaviour among children. *Nutrients* 2015;7:8565-8576. |
| S61 | Giskes K, van Lenthe F, Avendano-Pabon M, Brug J. A systematic review of environmental factors and obesogenic dietary intakes among adults: are we getting close to understanding obesogenic environments? *Obesity Reviews* 2010;12:e95-e106. |
| S62 | Wall J, Mhurchu CN, Blakely T, Rodgers A, Wilton J. Effectiveness of monetary incentives in modifying dietary behaviour: A review of randomised controlled trials. *Nutrition Reviews* 2006;64(12):518-531. |
| S63 | Williams J, Scarborough P, Matthews A, Cowburn G, Foster C, Roberts N, Rayner M. A systematic review of the influence of the retail food environment around schools on obesity-related outcomes. *Obes Rev* 2014;15:359-374. |
| S64 | Kent JL, Thompson S. The three domains of urban planning for health and well-being. *Journal of Planning Literature* 2014;1-18. |
| S65 | Delgado-Noguera M, Tort S, Martinez-Zapata MJ, Bonfill X. Primary school interventions to promote fruit and vegetable consumption: A systematic review and meta-analysis. *Preventive Medicine* 2011;53:3-9. |
| S66 | Feng J, Glass TA, Curriero FC, Stewart WF, Schwartz BS. The built environment and obesity: A systematic review of the epidemiologic evidence. *Health &Place* 2010;16:175-190. |
| S67 | De Vet E, De Ridder DTD, De Wit JBF. Environmental correlates of physical  activity and dietary behaviours among young people: A systematic review of reviews.  *Obes Rev* 2010;12:e130-e142. |
| S68 | Gamba RJ, Schuchter J, Rutt C, Seto EYW. Measuring the food environment and its effects on obesity in the United States: A systematic review of methods and results. *J Community Health* 2015;40:464-475. |
| S69 | Ganann R, Fitzpatrick-Lewis D, Ciliska D, Peirson LJ, Warren RL, Fieldhouse P,  Delgado-Noguera MF, Tort S, Harris SP, Martinez-Zapata MJ, Wolfenden L. Enhancing nutritional environments through access to fruit and vegetables in schools and homes among children and youth: A systematic review. *BMC Research Notes* 2014;7:422. |
| S70 | Atkinson RW, Kang S, Mills IC, Walton HA. Epidemiological time series studies of PM2.5 and daily mortality and hospital admissions: A systematic review and meta-analysis. *Thorax* 2014;0:1-6. |
| S71 | Balti EV, Echouffo-Tcheugui JB, Yako YY, Kengne AP. Air pollution and risk of type 2 diabetes mellitus: A systematic review and meta-analysis. *Diabetes Research and Clinical Practice* 2014;106:161-172. |
| S72 | Boothe VL, Boehmer TK, Wendel, AM, Yip FY. Residential traffic exposure and childhood leukemia: a systematic review and meta-analysis. *American Journal of*  *Preventive Medicine* [online] 2014;46(4):413-422. |
| S73 | CABE. Future health: Sustainable places for health and wellbeing. 2009. CABE: London, UK. Accessed 09 August 2016 from <http://webarchive.nationalarchives.gov.uk/20110118095356/http:/www.cabe.org.uk/> |
| S74 | Chen EK, Zmirou-Navier D, Padila C, Deguen S. Effects of air pollution on the risk of congenital anomalies: A systematic review and meta-analysis. *Int J Environ Res Public Health* 2014;11:7642-7668. |
| S75 | Deng Z, Chen F, Zhang M, Lan L, Qiao Z, Cui Y, An, J, Wang N, Fan Z, Zhao X, Li X. Association between air pollution and sperm quality: A systematic review and meta-analysis. *Environmental Pollution* 2016;208:663-669. |
| S76 | Eze IC, Hemkens LG, Bucher HC, Hoffman B, Schindler C, Kunzli N, Schikowski T, Probst-Hensch NM.. Association between ambient air pollution and diabetes mellitus in Europe and North America: Systematic review and meta-analysis. Environmental Health Perspectives, 123, 381-389. |
| S77 | Favarato G, Anderson H, Atkinson R, Fuller G, Mills I, Walton H. Traffic-related pollution and asthma prevalence in children. Quantification of associations with nitrogen dioxide. *Air Qual Atmos Health* 2014;7(4):459-466. |
| S78 | Hamra GB, Laden F, Cohen AJ, Raaschou-Nielsen O, Brauer M, Loomis D. Lung cancer and exposure to nitrogen dioxide and traffic: A systematic review. *Environmental Health Perspectives* 2014;123(11):1107-1112. |
| S79 | Hamra GB, Guha N, Cohen A, Laden F, Raaschou-Nielsen O, Samet JM, Vineis P, Forastiere F, Saldiva P, Yorfuji T, Loomis D. Outdoor particulate matter exposure and lung cancer: A systematic review and meta-analysis. *Environmental Health Perspectives* 2015;122(9):906-911. |
| S80 | Jafta N, Jeena PM, Barregard L, Naidoo RN. Childhood tuberculosis and exposure to indoor air pollution: a systematic review and meta-analysis. *The International Journal of Tuberculosis and Lung Disease* 2015;19(5):596-602. |
| S81 | Janghorbani M, Momeni F, Mansourian M. Systematic review and meta-analysis of air pollution exposure and risk of diabetes. *European Journal of Epidemiology* 2014;29(4):231-242. |
| S82 | Lacasaña M, Esplugues A, Ballester F.Exposure to ambient air pollution and prenatal and early childhood health effects. *European Journal of Epidemiology [online]* 2005;20(2):183-199. |
| S83 | Lui X, Lian H, Ruan Y, Liang R, Zhao X, Routledge M, Fan Z. Association  of exposure to particular matter and carotid intima-media thickness: A systematic  review and meta-analysis. *Int J Environ Res Public Health* 2015;12(10):12924-12940. |
| S84 | Luo C, Zhu X, Yao C, Hou L, Zhang J, Cao J, Wang A. Short-term exposure to particulate air pollution and risk of myocardial infarction: a systematic review and meta-analysis. *Environmental Science and Pollution Research* 2015;22(19):14651-14662. |
| S85 | Mehta S, Shin H, Burnett R, North T, Cohen AJ. Ambient particulate air pollution and acute lower respiratory infections: a systematic review and implications for estimating the global burden of disease. *Air Quality, Atmosphere & Health* 2013;6(1):69- 83. |
| S86 | Najafi TF, Roudsari RL, Namvar F, Ghanbarabadi VG, Talasaz ZH, Esmaeli M. Air Pollution and Quality of Sperm: A Meta-Analysis. *Iranian Red Crescent Medical Journal* 2015;17(4). |
| S87 | Pedersen M, Stayner L, Slama R, Figueras F, Nieuwenhuijsen MJ, Dadvand P. Ambient air pollution and pregnancy-induced hypertensive disorders: A systematic review and meta-analysis. *Hypertension* 2014;64(3):494-500. |
| S88 | Rodriguez-Villamizar LA, Magico A, Osornio-Vargas A, Rowe BH. The effects of outdoor air pollution on the respiratory health of Canadian children: A systematic review of epidemiological studies. *Canadian Respiratory Journal* 2015;22(5):282-  292. |
| S89 | Scheers H, Jacobs L, Casas L, Nemery B, Nawrot TS. Long-Term Exposure to Particulate Matter Air Pollution Is a Risk Factor for Stroke Meta-Analytical Evidence. *Stroke* 2015;46(11):3058-3066. |
| S90 | Shah AS, Lee KK, McAllister DA, Hunter A, Nair H, Whiteley W, Langrish JP, Newby DE, Mills NL. Short term exposure to air pollution and stroke: systematic review and meta-analysis. *BMJ* 2015;350:h1295. |
| S91 | Song Q, Christiani DC, Ren J. The global contribution of outdoor air pollution to the incidence, prevalence, mortality and hospital admission for chronic obstructive pulmonary disease: a systematic review and meta-analysis. *International Journal of Environmental Research and Public Health* 2014;11(11):11822-11832. |
| S92 | Teng THK, Williams TA, Bremner A, Tohira H, Franklin P, Tonkin A, Jacobs I, Finn J. A systematic review of air pollution and incidence of out-of-hospital cardiac arrest. *Journal of Epidemiology and Community Health* 2014;68(1):37-43. |
| S93 | Turner MC, Wigle DT, Krewski D. Residential pesticides and childhood leukemia: a systematic review and meta-analysis. *Ciencia & saude coletiva* 2011;16(3):1915- 1931. |
| S94 | Vrijheid M, Martinez D, Manzanares S, Dadvand P, Schembari A, Rankin J, Nieuwenhuijsen M. Ambient air pollution and risk of congenital anomalies: a systematic review and meta-analysis. *Environmental Health Perspectives* 2011;119(5):598. |
| S95 | Bonzini M, Carugno M, Grillo P, Mensi C, Bertazzi PA, Pesatori AC. Impact of ambient air pollution on birth outcomes: systematic review of the current evidences. *La Medicina del lavoro* 2009;101(5):341-363. |
| S96 | Frutos V, Gonzalez-Comadran M, Sola I, Jacquemin B, Carreras R, Checa Vizcalino MA. Impact of air pollution on fertility: A systematic review. *Gynaecological Endocrinology* 2015;31(1):7-13. |
| S97 | Hu H, Ha S, Roth J, Kearney G, Talbott EO, Xu X. Ambient air pollution and hypertensive disorders of pregnancy: A systematic review and meta-analysis. *Atmospheric Environment* 2014;97:336-345. |
| S98 | Koranteng S, Vargas ARO, Buka I. Ambient air pollution and children’s health: A systematic review of Canadian epidemiological studies. *Paediatrics & Child Health* 2007;12(3):225-233. |
| S99 | Stieb DM, Chen L, Eshoul M, Judek S. Ambient air pollution, birth weight and preterm birth: a systematic review and meta-analysis. *Environmental Research [online]* 2012;117:100-111. |
| S100 | Peters R, Peters J, Booth A, Mudway I. Is air pollution associated with increased risk of cognitive decline? A systematic review. *Age Ageing* 2015;44(5):755-760. |
| S101 | Vienneau D, Schindler C, Perez L, Probst-Hensch N, Röösli M. The relationship between transportation noise exposure and ischemic heart disease: a meta-analysis. *Environmental Research* 2015;138:372-380. |
| S102 | Waite T, Murray V, Baker D. Carbon Monoxide Poisoning and Flooding: Changes in Risk Before, During and After Flooding Require Appropriate Public Health Interventions Version 1. *PLoS Curr* 2014;3;6. |
| S103 | Ahern M, Kovats RS, Wilkinson P, Few R, Matthies F. Global health impacts of floods: Epidemiologic evidence. *Epidemiologic Reviews* 2005;27:36-46. |
| S104 | Alderman K, Turner LR, Tong S. Floods and human health: A systematic  review. *Environment International* 2012;47:37-47. |
| S105 | Stanke C, Murray V, Amlot R, Nurse J, Williams R. The effects of flooding on mental health: Outcomes and recommendations from a review of the literature. *PLoS Current Disasters* 2012;doi: 10.1371/4f9f1fa9c3cae. |
| S106 | Gascon M, Triguero-Mas M, Martinez D, Dadvand P, Forus J, Plasencia A, Nieuwenhuijsen MJ. Mental health benefits of long-term exposure to residential green and blue spaces: A systematic review. *J Environ Res Public Health* 2015;12:4354-4379. |
| S107 | Hunter RF, Christian H, Veitch J, Astell-Burt T, Hipp JA, Schipperijn J. The impact of interventions to promote physical activity in urban green space: a systematic review and recommendations for future research. *Social Science & Medicine* 2015;124:246-256. |
| S108 | Lee ACK, Maheswaran R. The health benefits of urban green spaces: A review of the evidence. *Journal of Public Health* 2010;33(2):212-222. |
| S109 | Thompson Coon J, Boddy K, Stein K, Whear R, Barton J, Depledge MH. Does participating in physical activity in outdoor natural environments have a greater effect on physical and mental wellbeing than physical activity indoors? A systematic review. *Environmental Science & Technology* 2011;45(5):1761-1772. |
| S110 | Bowler DE, Buyung-Ali L, Knight TM, Pullin AS. Urban greening to cool towns and cities: A systematic review of the empirical evidence. *Landscape and Urban Planning* [online] 2010;97(3):147-155. |
| S111 | Davison KK, Lawson CT. Do attributes in the physical environment influence children's physical activity? A review of the literature. *IBNPA* 2006;3(1):19. |
| S112 | Rothman L, Buliung R, Macarthur C, To T, Howard A. Walking and child pedestrian injury: a systematic review of built environment correlates of safe walking. Injury Prevention: *Journal of the International Society for Child and Adolescent Injury Prevention* 2014;20(1):41-49. |
| S113 | Cairns J, Warren J, Garthwaite K, Greig G, Bambra C. Go slow: an umbrella review of the effects of 20 mph zones and limits on health and health inequalities. *Journal of Public Health (Oxford, England)* 2015;37(3):515-520. |
| S114 | Aeron-Thomas A, Hass S. Red-light cameras for the prevention of road traffic  crashes. *Cochrane Database of Systematic Reviews* 2005;doi: <https://doi.org/10.1002/14651858.CD003862.pub2>. |
| S115 | Høye A. Speed cameras, section control, and kangaroo jumps–a meta-analysis.  *Accident Analysis & Prevention* 2014;73:200-208. |
| S116 | Pilkington P, Kinra S. Effectiveness of speed cameras in preventing road traffic collisions and related casualties: A systematic review. BMJ 2005;330:doi:  <https://doi.org/10.1136/bmj.38324.646574.AE>. |
| S117 | Calogiuri G, Chroni S. The impact of the natural environment on the promotion of active living: An integrative systematic review. *BMC Public Health* 2014;14:873. |
